# Absence among National Health Service workers during the COVID-19 pandemic in Portugal: Joana Alves

**DOI:** 10.1093/eurpub/ckac130.024

**Published:** 2022-10-25

**Authors:** J Alves, C Nunes

**Affiliations:** 1 Public Health Research Centre, NOVA National School of Public Health, Lisbon, Portugal; 2 Comprehensive Health Research Centre, NOVA National School of Public Health, Lisbon, Portugal; 3 Directorate-General of Health, Lisbon, Portugal

## Abstract

**Background:**

By March 2020, the first Covid-19 cases were detected in Portugal. While the National Health Service (NHS) faced an increased demand for health care, anecdotal evidence showed that the NHS absenteeism rose. This might be explained by outbreaks in healthcare units, COVID-19 infection due to close contact with patients, self-isolation and quarantines, and family challenges originated by lockdowns. The present work aimed to quantify the absenteeism among NHS workers during the COVID-19 pandemic in Portugal.

**Methods:**

This study used data for the number of NHS workers and absence days (2015-2021), from the Portuguese NHS Transparency Portal and the Strategy and Planning Office. Absenteeism was compared, before and after the pandemic onset, in absolute terms, and as absence rates (number of absent days as a percentage of potential workforce days). Additionally, we performed an interrupted time series analysis, by fitting a Poisson regression model with level change. We controlled for data seasonality using Fourier terms (pairs of sine and cosine functions).

**Results:**

From 2015 until March 2020, the average monthly absence rate was of 12.2, rising to 14.4 in the remaining period. This represented an increase of 18% in the absence rate. The interrupted time series showed an increase of 10.8% in the NHS absenteeism after the pandemic onset [Relative risk =1.10; 95% confidence interval (CI) 1.10-1.11; p < 0.01]. When accounting for seasonality in the data, the model showed an increase of 11.0% in the NHS absenteeism [Relative risk =1.11; 95% CI 1.01-1.22; p < 0.05].

**Conclusions:**

These results highlight the excess of absence days among the NHS workers during the COVID-19 pandemic. In future healthcare crises, health professionals should be protected, by assuring a safe workplace and making protective equipment available. Only then will be possible to reduce constraints in healthcare assistance, guarantee the adequate response, and contain the absence costs.

**Key messages:**

- During the COVID-19 pandemic in Portugal, the NHS absenteeism increased by 11% (p < 0.05).
- The absence rates might have threatened healthcare assistance, and increased the healthcare costs.

